# Development of Occupational Health Measures for the National Health Security Preparedness Index

**DOI:** 10.3389/fpubh.2016.00079

**Published:** 2016-04-25

**Authors:** Olaniyi O. Olayinka, Muge Akpinar-Elci

**Affiliations:** ^1^Center for Global Health, College of Health Sciences, Old Dominion University, Norfolk, VA, USA

**Keywords:** disaster, national health security, metrics, indicators, preparedness, occupational health and safety

The 2001 World Trade Center and 2005 Hurricane disasters, and the 2014 Ebola outbreak were major events that tasked the United States’ public health emergency preparedness and response apparatus. The health and economic cost of these events is huge including over 4000 deaths and damages to infrastructure worth hundreds of billions of dollars. Unfortunately, the U.S. labor force was disproportionately affected (1–4). Similar public health impact of other disasters on workers has also been reported. In the immediate aftermath of the Gulf of Mexico oil spill, for instance, approximately 75% of those who developed acute health effects and sought medical care were clean-up workers (5).

The United States civilian labor force is large (ranks third globally), with approximately 160 million people, and most disasters tend to occur during work hours (6–8). Hence, we believe health security strategies that are customized to the needs of workers are critical. Additionally, we believe future evaluation of national preparedness and response level should consider objective occupational health and safety metrics.

Since the Pandemic and All-Hazards Preparedness Reauthorization Act was passed in 2006, the Department of Health and Human Services (DHHS) has engaged in efforts to improve the health security of the nation (9). To date, two National Health Security Strategies (NHSS) and implementation plans (2010–2014 and 2015–2018) have been developed (10, 11). More importantly, DHHS recently completed its first quadrennial evaluation of the progress made in achieving its national health security goals – the National Health Security Review 2010–2014 (12). Despite the progress that has been made in achieving its goals, the review recognizes that “the science of evaluation in this emerging area of expertise is still relatively new.”

However, evidence-based evaluation of national public health programs is critical to their future success (13). Hence, the collaboratively developed National Health Security Preparedness Index (NHSPI), inspired by the Centers for Disease Control and Prevention, is welcomed (14). To the best of our knowledge, the NHSPI is the only tool currently available to measure the preparedness of each of the states in the U.S. The index is calculated based on values assigned to 194 measures that are believed to influence six broad domains of national health security (15, 16). The domains are (i) incident and information management, (ii) health-care delivery, (iii) environmental and occupational health, (iv) countermeasure management, (v) community planning and engagement, and (vi) health security surveillance. On reviewing the environmental and occupational health domain of the index, we found that only measures of environmental health were included; there are no indicators of occupational health and safety (17).

As the NHSPI gets revised in the future, we hope objective measures of occupational health and safety would be considered. We believe collaboration with government and academic institutions that have experience developing OHS metrics could be beneficial. For instance, there exist a number of federal and state initiatives targeting the safety and health of emergency responders (18–23). We provide below a brief review of some of the efforts to improve worker’s safety and health, and our suggestion on their usefulness as potential sources of OHI:

1. The Council of State and Territorial Epidemiologists (CSTE) – occupational health indicators (OHIs) (18):
  - CSTE and the National Institute of Occupational Safety and Health (NIOSH) recently developed 22 OHIs.
  - Some of the CSTE-OHIs, especially for those that state-level data are available, could be validated and considered for possible incorporation into the NHSPI. For instance, exhaustive data on the rate of work-related fatalities, non-fatal injuries, and illnesses from most US’ States and Territories are collected by the United States Department of Labor and are accessible online (24).
2. NIOSH – Fatality Assessment and Control Evaluation (FACE) Program (19):
  - The FACE program was developed by NIOSH in the 1980s and surveils risk factors for occupational fatalities. However, States’ participation in the program is voluntary. Because of its goal to prevent occupational fatalities, using surveillance data, one could argue that the FACE program is critical to the safety of workers, especially for young workers who account for the majority of reported work-related fatalities (25).
  - Each State’s effective participation in the FACE program, or similar program, could be considered an indication of their commitment to the safety and health of workers.
3. NIOSH – Fire Fighter Fatality Investigation and Prevention Program (FFFIPP) (20):
  - This is another NIOSH program that investigates work-related deaths, specifically among fire fighters in the United States. State-level data are reported on the program’s website. The mortality data obtained from this program are used to develop recommendations to fire departments with the aim of preventing future deaths and injuries. Because firefighters play a major role in disaster and emergency response, it is important that fire departments abide by FFFIPP’s recommendations.
  - Hence, data reported from the program, including firefighter fatality rate and implementation of recommendations from NIOSH investigations, could possibly serve as indicators of firefighter safety and health.
4. Training programs and Educational Resources for Emergency Responders including the
  - Guidance Documents for Protecting Emergency Responders (21): This guidance document was jointly prepared by NIOSH and the RAND Corporation. Consisting of four volumes, the documents are educational resources for emergency responders covering issues related to personal protective equipment and personal safety of responders.
  - Emergency Responder Health Monitoring and Surveillance Program (22): This NIOSH program has published a comprehensive framework for protecting and monitoring the health of emergency responders. The document is relevant for training and use by all levels of personnel involved in emergency response activities.
  - The Disaster Site Worker Outreach Training Program (23): This training program was developed by Occupational Safety & Health Administration (OSHA) and required for those who plan to work on disaster sites. Specifically, trainees learn to identify, control, and prevent themselves from hazards on the disaster work site.

Overall, measures of the level and frequency of safety and health training that are available to emergency professionals and responders in each state could be considered for inclusion in the NSHPI.

In conclusion, whichever occupational health measure or indicator gets selected for the NSHPI should also be one that (1) represents the general workforce and emergency responders, (2) can be assessed at the state level, and (3) is easy to obtain. These are in addition to the rigorous and practical selection criteria that are being used in developing the NHSPI. The ultimate goal is to have OHIs that truly reflect the level of preparedness of each state to respond to a broad range of disasters and emergency situations.

## Author Contributions

MA-E conceived of the manuscript. OO drafted the manuscript. Both OO and MA-E read, revised, and approved of the manuscript.

## Conflict of Interest Statement

The authors declare that the research was conducted in the absence of any commercial or financial relationships that could be construed as a potential conflict of interest.
